# An angiogenesis‐related long noncoding RNA signature correlates with prognosis in patients with hepatocellular carcinoma

**DOI:** 10.1042/BSR20204442

**Published:** 2021-04-07

**Authors:** Dengliang Lei, Yue Chen, Yang Zhou, Gangli Hu, Fang Luo

**Affiliations:** 1 Department of Hepatobiliary Surgery, The First Affiliated Hospital of Chongqing Medical University, Chongqing 400016, China; 2 Central Laboratory, The First Affiliated Hospital of Chongqing Medical University, Chongqing 400016, China

**Keywords:** angiogenesis, GSEA, mRNA signature

## Abstract

Hepatocellular carcinoma (HCC) is one of the most prevalent and lethal cancers worldwide. Neovascularization is closely related to the malignancy of tumors. We constructed a signature of angiogenesis-related long noncoding RNA (lncRNA) to predict the prognosis of patients with HCC. The lncRNA expression matrix of 424 HCC patients was downloaded from The Cancer Genome Atlas (TCGA). First, gene set enrichment analysis (GSEA) was used to distinguish the differentially expressed genes of the angiogenesis genes in liver cancer and adjacent tissues. Next, a signature of angiogenesis-related lncRNAs was constructed using univariate and multivariate analyses, and receiver operating characteristic (ROC) curves were used to assess the accuracy. The signature and relevant clinical information were used to construct the nomogram. A 5-lncRNA signature was highly correlated with overall survival (OS) in HCC patients and performed well in evaluations using the C-index, areas under the curve, and calibration curves. In summary, the 5-lncRNA model can serve as an accurate signature to predict the prognosis of patients with liver cancer, but its mechanism of action must be further elucidated by experiments.

## Introduction

Liver cancer is considered one of the most common cancers worldwide [1], and HCC is the most common cause of liver cancer [2]. Despite advances in surgical and ablative therapies, the high recurrence and metastasis of liver cancer remain the leading causes of all cancer deaths [3,4]. Additionally, an increase in nonalcoholic fatty liver disease (NAFLD), metabolic syndrome and obesity increases the risk of liver cancer [5]. Therefore, identifying new predictors to improve the prognosis of HCC is urgent.

Tumor angiogenesis plays a crucial role in the occurrence and development of HCC; thus, tumor blood vessels are one of the key targets of tumor therapy management [6]. Because of the high proliferation of tumors, tumors must induce massive angiogenesis to meet their needs [7]. Neovascularization shows immaturity and high heterogeneity [6], leading to decreased immune cell infiltration and activity and an increased risk of metastatic disease [8,9]. Thus, it is critical to identify angiogenesis-related biomarkers that serve as valuable early diagnostic and prognostic biomarkers for HCC patients.

Long noncoding RNAs are noncoding RNAs with a length greater than 200 nt that play a critical role in regulating cell differentiation, growth and development [10]. In recent years, increasing evidence has shown that lncRNAs are related to various cancers [11]. Additionally, lncRNAs are closely related to tumor occurrence, metastasis and tumor stage [12–14]. For example, HOTAIR, as a carcinogenic lncRNA, is highly expressed in breast cancer, gallbladder cancer and other cancers, is associated with invasion and metastasis and is a diagnostic and prognostic indicator for various cancers [15–19]. However, studies on the characteristics of angiogenesis-related lncRNAs during HCC survival are still lacking.

In the present study, we developed and verified an angiogenesis-related lncRNA signature to predict the OS of patients with HCC for the first time. Additionally, we further explored the relationship between the five lncRNAs and HCC progression. A nomogram based on five lncRNA characteristics and clinical factors was then used to assess clinical significance. GSEA was used to identify potential biological processes and molecular mechanisms, such as apoptosis, autophagy and the cell cycle. Finally, we analyzed the relationship between the model and immune cells. In summary, we found that the lncRNA signature not only serves as independent prognostic factor for HCC but also has better predictive ability for HCC patient survival than existing lncRNA-related signatures.

## Materials and methods

### Data collection

The lncRNA expression data and clinical information were downloaded from the TCGA database (https://portal.gdc.cancer.gov/) and were randomly divided into a training set and verification set at a 3:1 ratio. Detailed information is shown in Table 1.

**Table 1:** Summary of baseline clinical pathological parameters of patients with HCC in the two datasets

Characteristic	Train	Test
Age (years)		
≤65	165	59
>65	87	32
Gender		
Male	170	64
Female	82	27
Grade		
1	31	14
2	126	42
3	87	31
4	8	4
Stage		
I	122	47
II	65	44
III	60	N/A
IV	5	N/A
T stage		
T1	123	48
T2	67	19
T3	57	19
T4	5	5
Survival status		
Alive	172	60
Deceased	80	31

Abbreviations: HCC, hepatocellular carcinoma; N/A, not applicable.

### Angiogenesis-related lncRNAs

GSEA was used to identify angiogenesi**s**-related gene sets (http://www.gsea-msigdb.org/gsea/msigdb/search.jsp) in 50 HCC tissues and their adjacent tissues. Finally, 123 genes related to angiogenesis were included in subsequent studies. The relationship between lncRNAs and angiogenesis genes was calculated based on their expression value. LncRNAs were selected for subsequent study based on a Spearman’s correlation coefficient absolute value >0.3 and *P*<0.01.

### Development of an angiogenesis-related lncRNA signature

In the training set, we first identified 864 angiogenesis-related lncRNAs. Univariate Cox regression analysis (*P*<0.05) was used to obtain 123 lncRNAs associated with prognosis. Finally, multivariate Cox regression analysis was used to construct a signature containing five lncRNAs, as detailed in Table 2. The risk score for each patient was calculated using the following equation:

$$\text{risk score}=\sum_{\left(i=1\right)}^{n}\;Coef(i)\times x(i),$$

where Coef (*i*) and *x*(*i*) represent the estimated regression coefficient and value of each lncRNA expression, respectively. All the patients were divided into high-risk and low-risk groups based on the median risk score. Kaplan–Meier survival curves and two-sided log-rank tests were used to compare the OS of different patients. ROC curves were applied to assess the diagnostic efficacy of each clinicopathological characteristic and prognostic signature. Stratified survival analysis was performed to examine the accuracy of the prognostic signature in predicting patient survival outcomes. Furthermore, we performed univariate and multivariate Cox regression analyses to evaluate whether the risk score was independent in determining the prognosis of HCC patients. *P*<0.05 was considered statistically significant.

**Table 2:** The information of five lncRNAs associated with overall survival in patients with HCC

LncRNA	Coef	HR	*P*-value	Risk
LINC01138	0.301906	1.35243	*P*=0.001	High
LINC00942	0.028688	1.02910	*P*<0.001	High
AL031985.3	0.350479	1.41974	*P*<0.001	High
AC015908.3	-0.155085	0.85634	*P*=0.004	Low
USP46-AS1	0.370322	1.44820	*P*=0.002	High

Abbreviation: HCC, hepatocellular carcinoma.

The lncRNA expression profile matrix of HCC patients from the verification set was used as independent validation for the accuracy of the 5-lncRNA constructed model.

### Establishment and validation of the nomogram

We constructed a nomogram by integrating traditional clinical variables such as age, stage and the risk score derived from the prognostic signature to analyze the probable 3- and 5-year OS of patients with HCC. We then used the concordance index (C-index) to evaluate the discriminative and predictive ability of the nomogram. Furthermore, calibration curves of the nomogram were generated to examine the concordance between the predicted survival and observed survival after bias correction.

### Construction of the lncRNA–mRNA coexpression network

A coexpression network was constructed to analyze the correlation between angiogenesi**s**-related lncRNAs and their target mRNAs. Pearson’s correlation coefficients were calculated to identify the mRNAs that were significantly associated with their target lncRNAs based on an absolute threshold coefficient value > 0.3.

### Gene set enrichment analysis (GSEA)

GSEA was used to identify angiogenesi**s**-related gene sets in 50 HCC tissues and their adjacent tissues. GSEA was also used to further analyze the underlying molecular mechanisms. The gene sets were filtered using the maximum and minimum gene set sizes of 500 and 15 genes, respectively. The enriched gene sets were obtained based on a *P* value < 0.05 and a false discovery rate (FDR) < 0.25 after performing 1000 permutations.

### Statistical analysis

The data were processed using the PERL programming language (http://www.perl.org/; Version 5.30.2). Statistical analyses were performed using GraphPad Prism 8.0 software or R software in a double-blind manner. *P*<0.05 was regarded as statistically significant.

## Results

### Identification of angiogenesis-related genes

**Figure 1:**
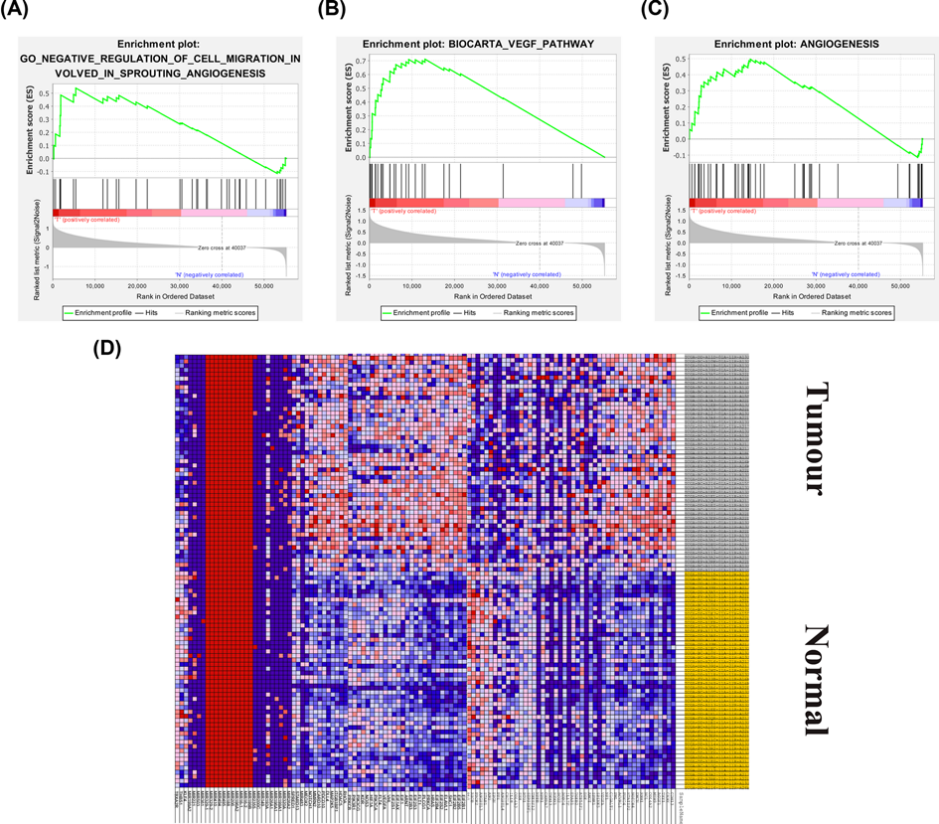
GSEA of starvation-related gene sets (**A–C**) Enrichment map of one angiogenesis-related gene set between liver cancer and paired adjacent tissues identified by GSEA. (**D**) Heat map of 123 genes in liver cancer and normal tissue.

GSEA was used to determine significant differences in the angiogenesis-related gene set between HCC and paired adjacent samples. The angiogenesis-related gene set was significantly enriched in HCC samples (Figure 1A–C). In total, 123 angiogenesis-related genes were used in the subsequent study (Supplementary File S1). The heat map shows differences in the expression of angiogenesis-related genes in hepatocellular carcinoma and adjacent tissues (Figure 1D).

### Identification of angiogenesis-related long noncoding RNAs

From GSEA, we collected 123 angiogenesis-related genes. Next, 864 angiogenesis-related lncRNAs were identified. Subsequently, five angiogenesis-associated lncRNAs, LINC01138, LINC00942, AL031985.3, AC015908.3 and USP46-AS1, were analyzed in the training set using univariate Cox regression analysis, Kaplan–Meier (KM) inspection and multivariate Cox regression analysis (Table 2).

### Construction and validation of a signature

**Figure 2:**
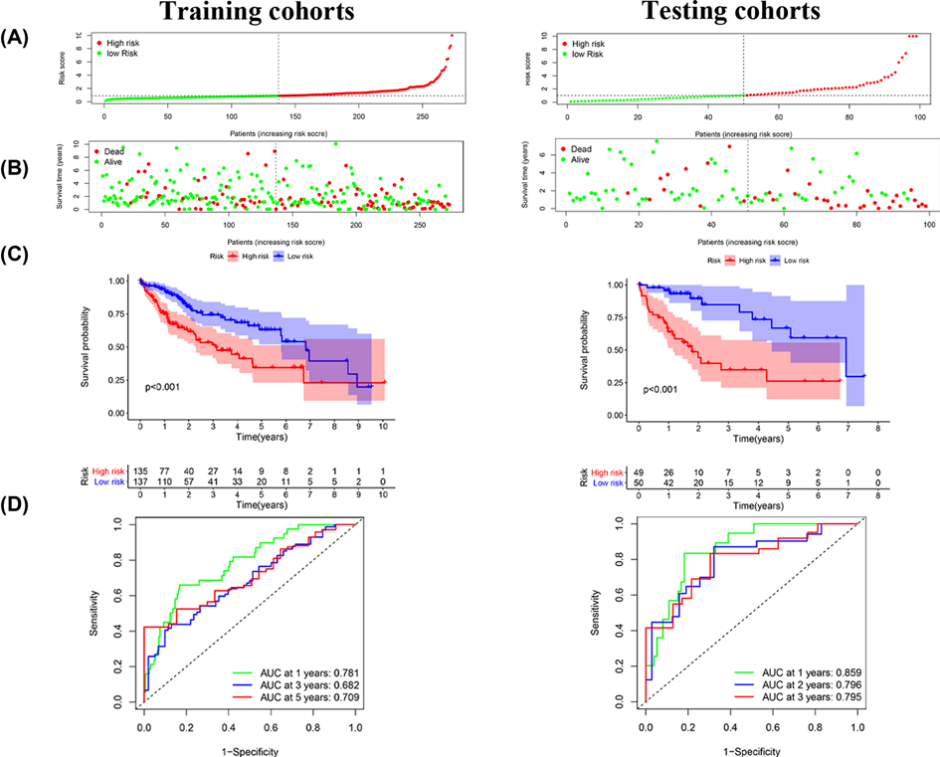
Construction and validation of the angiogenesis-related lncRNA prognostic signature in patients with HCC (**A** and **B**) The distribution and scatter plots of risk scores based on angiogenesis-related lncRNA prognostic characteristics of high- and low-risk HCC patients show the correlation between survival time and risk scores based on the angiogenesis-related lncRNA prognostic characteristics of HCC patients. (**C**) The KM curves show that the survival time of patients with low risk scores based on the angiogenesis-related lncRNA prognostic signature is significantly longer than that of patients with high-risk scores. (**D**) Time-dependent ROC curves at 1, 3 and 5 years show the accuracy of the signature in predicting the survival times (prognosis) of HCC patients from the TCGA database.

We combined these five angiogenesis-related lncRNAs to generate a signature to predict the clinical characteristics of HCC. The risk scores of each HCC case indicated that the patients in the high-risk group generally had a worse prognosis in the training set and testing cohort (Figure 2A,B). Additionally, the log-rank test and Kaplan–Meier survival curve analysis showed that patients with a low risk score had a much longer survival than those with a high risk score in the two cohorts (Figure 2C). The time-dependent ROC curves were analyzed to further assess the accuracy of the 5-angiogenesis-related lncRNA signature in predicting the survival of HCC patients at 3 and 5 years (Figure 2D). The time-dependent ROC curve showed that the AUC at 3 and 5 years were more than 0.682 in the training set and the validation cohort, respectively, indicating the signature had an excellent capacity to predict HCC patient survival. Next, we performed KM analysis for each lncRNA in the model. When five lncRNAs were used as a single biomarker, their diagnostic significance was significantly lower than that of ten angiogenesis-related lncRNAs, further indicating the accuracy of our signature (Supplementary File S2).

### Our signature correlates with disease progression

**Figure 3:**
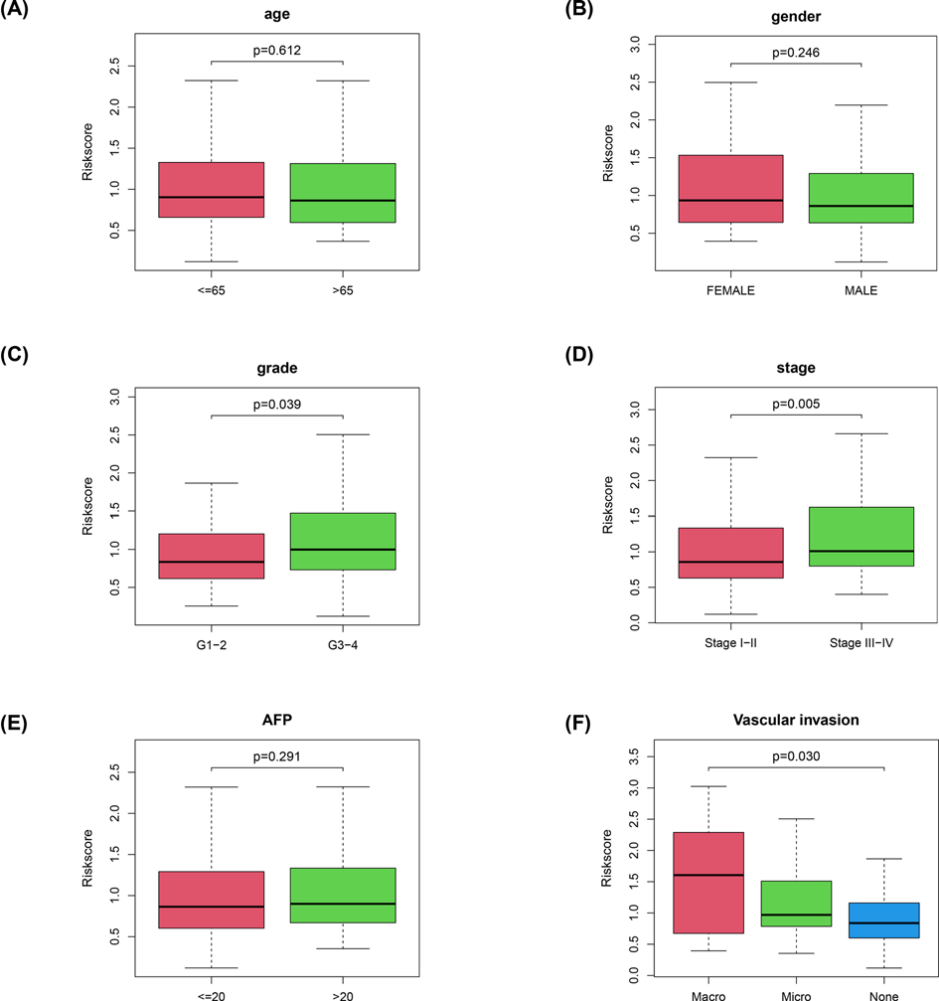
Correlation of our signature with the clinicopathological characteristics of HCC (**A**) Age (≥ 65 vs. <65 years; *P*=0.4375); (**B**) Sex (male vs. female; *P*=0.920); (**C**) Tumor grade (grade 1-2 vs. 3-4; *P*=0.032); (**D**) Tumor stage (stage 3-4 vs. 1-2; *P*=0.005); (**E**) Alpha-fetoprotein(AFP) (≤20 vs. >20 ng/ml; *P*=0.291). (**F**) Vascular invasion (none vs. micro vs. macro; *P*=0.030).

In the training cohort, we assessed the relationship between the signature and HCC clinicopathological characteristics (Figure 3A–F). The result reveals that our signature was significantly associated with grade, stage and vascular invasion, suggesting that our signature plays a crucial role in HCC progression.

### The signature acts as an independent prognostic indicator

**Figure 4:**
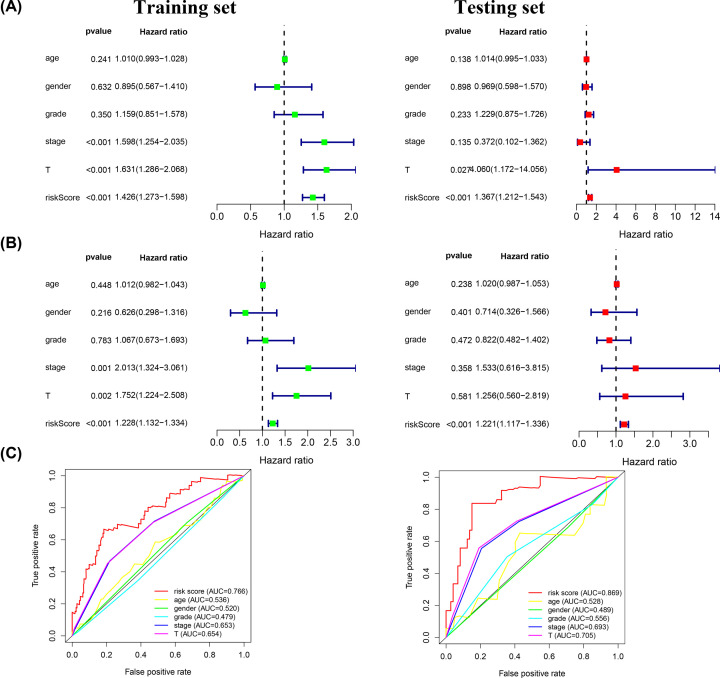
Estimation of the prognostic accuracy of the signature and other clinicopathological variables (**A** and **B**) In the training and validation sets, univariate and multivariate analyses were performed for risk scores and each clinical feature. (**C**) The time-dependent ROC curves of risk scores and clinical features were predicted in the training and validation sets at 5 years.

Cox regression analysis was used to determine whether the 5-angiogenesis-related lncRNA signature was an independent prognostic factor for HCC. Age, sex, grade, stage and T stage were included as clinical factors (Figure 4A,B). Univariate analysis revealed that stage, T stage and the risk score were associated with survival in the training set, and T stage and risk score were associated with survival in the validation cohort. Additionally, multivariate Cox analysis showed that T stage and the risk score were associated with survival in the training set, and the risk score was associated with survival in the validation cohort. Thus, our signature could be an independent prognostic factor in HCC. The AUC values in the training and validation sets were 0.766 and 0.869, respectively, indicating high accuracy of the risk score as an independent prognostic factor (Figure 4C).

**Figure 5:**
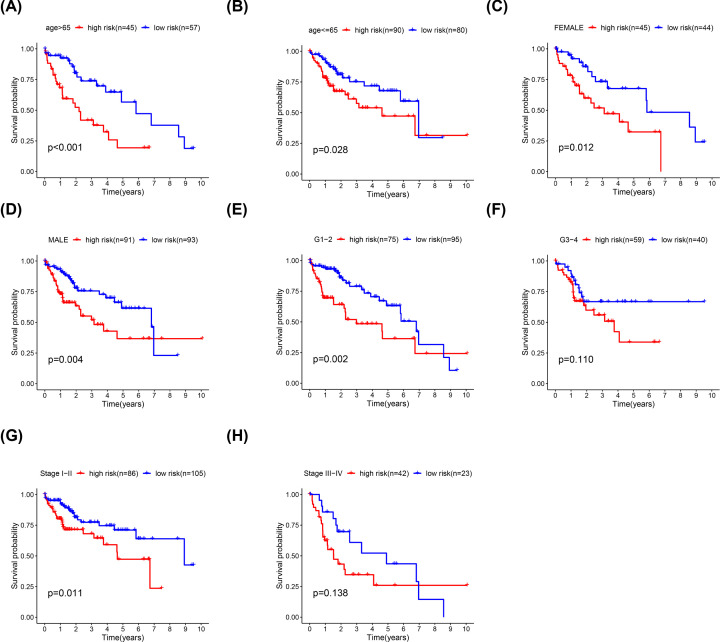
Survival rates of high- and low-risk HCC patients stratified by different clinicopathological characteristics (**A–H**) Kaplan–Meier survival curve analysis shows the overall survival (OS) rates of high- and low-risk HCC patients from the TCGA database stratified by age (≤65 vs. >65 years), sex (male vs. female), tumor grade (high grade vs. low grade), stage (stages I and II vs. stages III and IV) and T stage (T1/T2 vs. T3/T4).

Stratified analysis was conducted according to age, sex, stage and T stage. Our risk stratification based on the angiogenesis-related lncRNA signature remains a powerful tool for predicting HCC survival by age, sex, tumor stage and T stage (Figure 5A–H).

### A nomogram based on the signature

**Figure 6:**
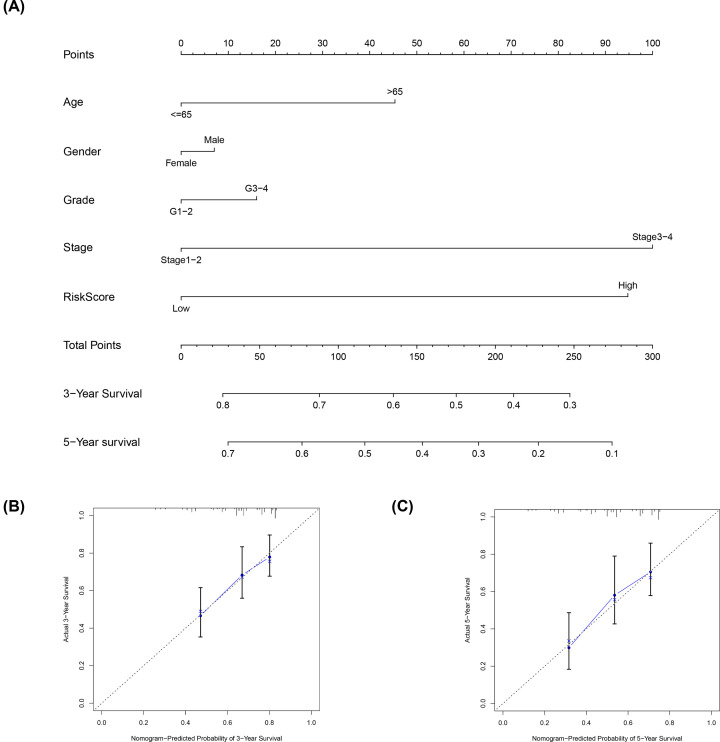
An established nomogram for predicting OS (**A**) Construction and validation of the prognostic nomogram with the starvation-related mRNA prognostic signature risk score as one of the parameters in the training set. (**B** and **C**) Calibration curves of the nomogram for the prediction of 3- and 5-year OS.

To provide clinicians with a practical clinical tool for predicting the 3- and 5-year OS incidence in patients with HCC, we constructed a nomogram based on the clinicopathological characteristics (age, sex, grade and stage) and risk score (Figure 6A). The 3- and 5-year OS calibration curves showed that the proposed nomogram had good predictive ability (Figure 6B,C).

### Analysis of biological processes associated with angiogenesis-related LncRNAs

**Figure 7:**
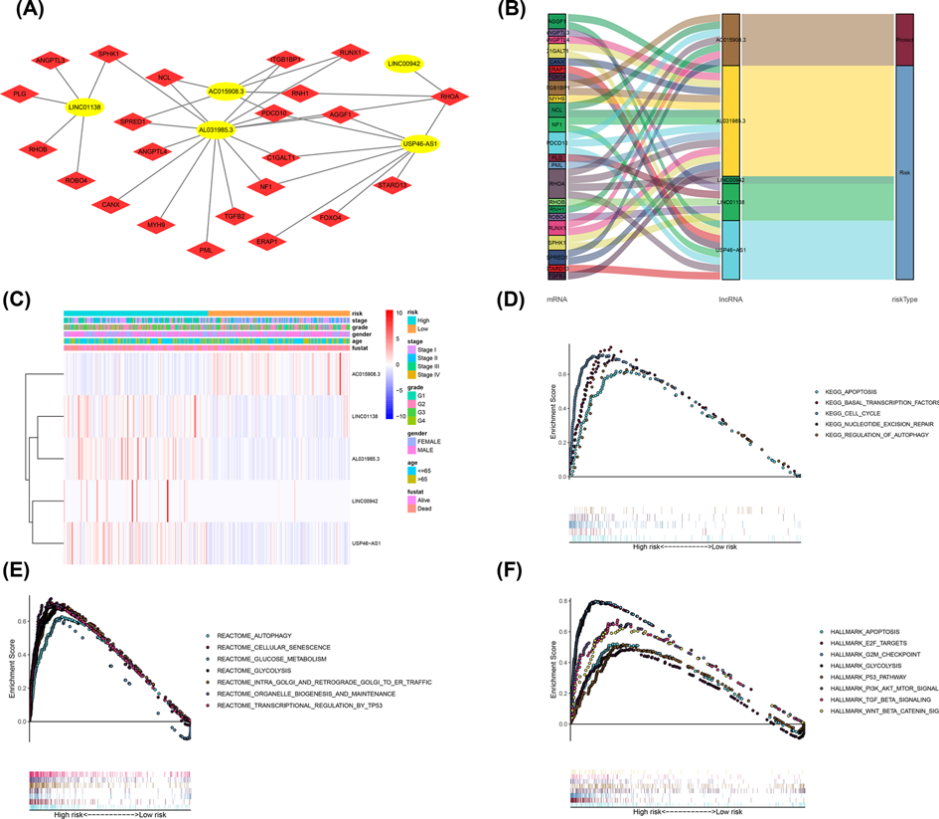
Construction of the angiogenesis-related lncRNA–mRNA coexpression network and functional enrichment analyses (**A**) Diagrammatic representation of the angiogenesis-related lncRNA–mRNA network shows 27 lncRNA–mRNA coexpression. The red circles correspond to angiogenesis-related lncRNAs, and the blue diamonds correspond to the mRNAs. (**B**) The Sankey diagram shows the connection degree between the 27 mRNAs and 5 angiogenesis-related lncRNAs (risk/protective). (**C**) Risk factor score, clinical features and expression of nine mRNAs in each patient. (**D–F**) Kyoto Encyclopedia of Genes and Genomes (KEGG) pathway, hallmark and Reactome analyses show the enriched signaling pathways associated with the high-risk group.

We investigated the potential functions of the five angiogenesis-related lncRNAs in HCC by constructing an lncRNA–mRNA coexpression network using Cytoscape. The lncRNA–mRNA coexpression network contained 27 pairs of lncRNA–mRNA (Pearson’s correlation coefficient |*R*| > 0.3 and *P*<0.05) (Figure 7A). Among these, 27 mRNAs were significantly correlated with the 5 lncRNAs in the prognostic signature. The Sankey diagram shows the relationship between the 27 mRNAs and 5 lncRNAs (risk/protective) (Figure 7B). The relationship between the lncRNA expression level and risk value and clinical factors are shown in Figure 7C. We conducted GSEA between the high-risk and low-risk groups to reveal the underlying molecular mechanisms of the angiogenesis-related lncRNA signature involved in HCC progression. KEGG analysis showed that apoptosis, the cell cycle and the regulation of autophagy in the high-risk group were the most significantly enriched pathways (Figure 7D). Additionally, the angiogenesis-related lncRNA signature regulated glucose metabolism, glycolysis, the P53 pathway and TGF-B signaling, showing that the signature was involved in important bioregulation (Figure 7E,F).

### Association between the angiogenesis-related lncRNA signature and immune cells

**Figure 8:**
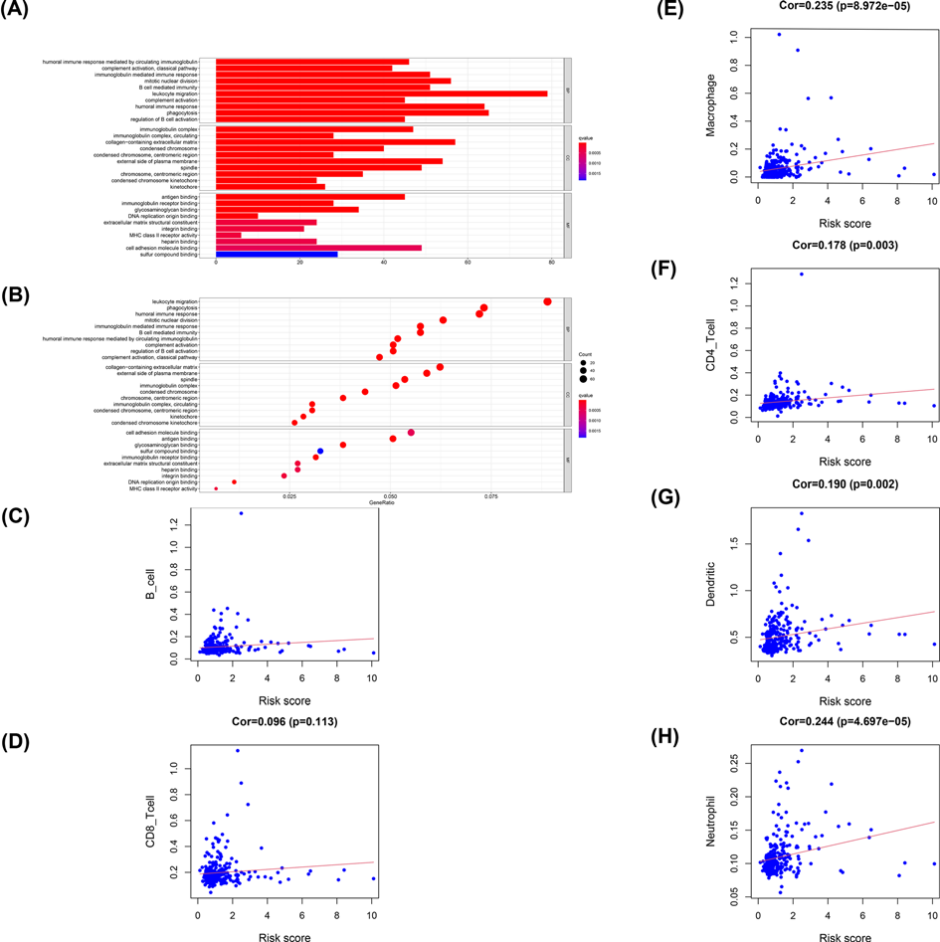
Correlation between signature and immune cells (**A** and **B**) GO analysis results showing the functions and enriched signaling pathways associated with the signature. (**C–H**) Correlation between immune cells and risk score.

Gene Ontology(GO) analysis enrichment analysis was used to further verify whether the signature was involved in immune response. As shown in Figure 8A,B, the most notable correlation in GO is related to immune response. Additionally, the signature was related to CD4-T cells, dendrites, macrophages and neutrophils but was independent of B cells and CD8-T cells. The correlation between macrophages was the highest, indicating an increased number of macrophages in the high-risk samples (Figure 8C–H). The above results suggest that our signature is associated with immune infiltration.

## Discussion

HCC is the second leading cause of cancer-related death globally, with approximately half of all cases and deaths occurring in China [20]. The high probability of metastasis and recurrence limits long-term survival [21]. Hyperproliferation of blood vessels and vascular abnormalities are common in liver cancer [22]. There is increasing evidence that angiogenesis is closely related to the malignancy of liver cancer [23]. Chen found that CPAP promotes angiogenesis and metastasis in hepatocellular carcinoma [24]. In pancreatic cancer, angiogenesis is an essential factor for tumor progression [25]. VEGF and VEGFRs, the most important and well-studied modulators of angiogenesis in recent years [26], play a crucial role in liver cancer [23]. In recent years, antiangiogenic therapy has been widely used in liver cancer treatment. As an antiangiogenic drug, sorafenib is used in the treatment of nonresectable or metastatic liver cancer. However, sorafenib only prolonged the average survival rate of HCC patients by 3 months because some patients exhibited sorafenib resistance [27–29]. Therefore, angiogenesis-related biomarkers are potential diagnostic biomarker and therapeutic targets for patients with HCC. In the present study, we show that 5-lncRNA signature analysis is an effective method to predict the prognosis of HCC patients independently. Additionally, patients with high angiogenesis-related risk scores had a poor prognosis. Notably, the risk score based on the 5-lncRNA signature was closely related to liver cancer development and immune cell infiltration.

Studies have found that lncRNAs play a crucial role in the malignant progression of HCC [30,31]. Additionally, lncRNAs are related to gene activation, autophagy, metabolism, inflammation, the immune response and other biological processes [32–35]. In particular, emerging evidence suggests that lncRNAs play a regulatory role in tumor angiogenesis [36]. For example, the long noncoding RNA UBE2CP3 can enhance VEGFA secretion by HCC cells and promote angiogenesis [37]. Recent studies have shown that angiogenesis-related lncRNAs may become a new therapeutic target and molecular marker for diseases, providing potential support for the clinical management and treatment of tumors [38,39]. Sun found that the 5-autophagy-related lncRNA signature accurately predicted the prognosis of BCLA patients [38]. Additionally, Luan established an autophagy-related lncRNA signature in glioma and advanced the targeted treatment of glioma [39]. Therefore, angiogenesis-related lncRNAs may become a new marker for liver cancer malignancy and a potential indicator of prognosis in patients with liver cancer.

In our study, we first identified 123 angiogenesis-related lncRNAs significantly related to OS by univariate Cox regression analysis of angiogenesis-related lncRNA expression in the samples of patients with liver cancer from the TCGA database. Additionally, five adaptive lncRNAs, LINC01138, LINC00942, AL031985.3, AC015908.3 and USP46-AS1, were selected according to the performance of multivariate Cox regression analysis to construct the prognostic signature. The risk score of each HCC patient was calculated based on the expression of five angiogenesis-related lncRNAs in the prognostic signature. Next, the patients were divided into high- and low-risk groups according to the median risk score. HCC patients with a low risk score had a better survival time than those with a high risk score for HCC. ROC curve analysis verified the accuracy of the prognostic features of angiogenesis-related lncRNAs in HCC patients. The risk score based on the angiogenesis-related lncRNA prognostic signature was an independent prognostic factor based on variable Cox regression analysis. Stratified correlation analysis showed that the prognostic features of angiogenesis-related lncRNAs accurately predicted survival in patients with high- and low-risk HCC. Five angiogenesis-related lncRNAs in HCC were further identified for their respective regulation, and an lncRNA–mRNA coexpression network was constructed. GSEA also revealed significant differences in angiogenesis-related signaling pathways between the high- and low-risk groups. Some angiogenesis-related pathways were enriched in the high-risk groups. Finally, we found an association between the signature based on genes associated with angiogenesis and immune cell infiltration. This finding suggests that enhanced immunity is associated with improved prognosis. These results are consistent with the notion that angiogenesis is a key regulator of HCC progression.

For the first time, an angiogenesis-related lncRNA signature was identified to be correlated with HCC progression and prognosis and could be used as independent prognostic molecular biomarkers for predicting HCC survival.

## Supplementary Material

Supplementary Files S1-S2

## Data Availability

All the data were obtained from The Cancer Genome Atlas (TCGA, https://portal.gdc.cancer.gov/) and Gene Set Enrichment Analysis (GSEA, http://www.gsea-msigdb.org/gsea/msigdb/search.jsp) databases.

## Abbreviations

GSEA: gene set enrichment analysis
HCC: hepatocellular carcinoma
lncRNA: long noncoding RNA
NAFLD: nonalcoholic fatty liver disease
OS: overall survival
ROC: receiver operating characteristic
TCGA: The Cancer Genome Atlas
