# Supplementary material for: An angiogenesis‐related long noncoding RNA signature correlates with prognosis in patients with hepatocellular carcinoma
Source: Biosci Rep. 2021 Apr 7;41(4):BSR20204442. doi: 10.1042/BSR20204442 (PMC8026853; doi:10.1042/BSR20204442)

Supplementary Materials

Supplementary File S1: Identification of 123 angiogenesis-related genes.

gene
ARNT EIF1 EIF1AX EIF2B1 EIF2B2 EIF2B3 EIF2B4 EIF2B5 EIF2S1 EIF2S2 EIF2S3 ELAVL1 FLT1 FLT4 HIF1A HRAS KDR NOS3 PIK3CA PIK3CG PIK3R1 PLCG1 PRKCA PRKCB SHC1 VEGFA VHL CARD10 DLL4 HDAC5 ITGB1BP1 KLF4 MAP2K5 MEOX2 MIR146A MIR149 MIR15A MIR16-1 MIR16-2 MIR188 MIR193A MIR196A1 MIR196A2 MIR199A1 MIR199A2 MIR199B MIR19B1 MIR19B2 MIR200C MIR206 MIR20A MIR22 MIR221 MIR2355 MIR26A1 MIR26A2 MIR29C MIR320A MIR329-1 MIR329-2 MIR361 MIR410 MIR424 MIR483 MIR494 MIR495 MIR497 MIR503 MMRN2 NOTCH1 PDCD10 RHOA SPRED1 STARD13 TBXA2R THBS1 ACVRL1 AGGF1 AMOT ANG ANGPTL3 ANGPTL4 ATP5IF1 BTG1 C1GALT1 CANX CDH13 CHRNA7 COL4A2 COL4A3 CXCL8 EGF EMCN EPGNERAP1 FOXO4 HTATIP2 IL17F IL18 MYH9 NCL NF1 NOTCH4 NPPB NPR1 PF4 PLG PML PROK2 RHOBRNH1 ROBO4 RUNX1 SCG2 SERPINF1 SHH SPHK1 SPINK5 STAB1 TGFB2 THY1 TNFSF12 TNNI3 VEGFA

Supplementary File S2: KM analysis of five lncRNAs. (A-E) Kaplan-Meier survival curve analysis shows the overall survival (OS) rates.

(A)

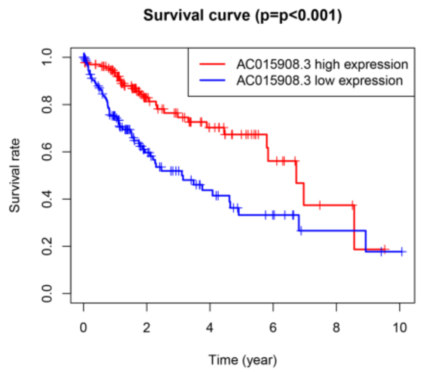

(B)

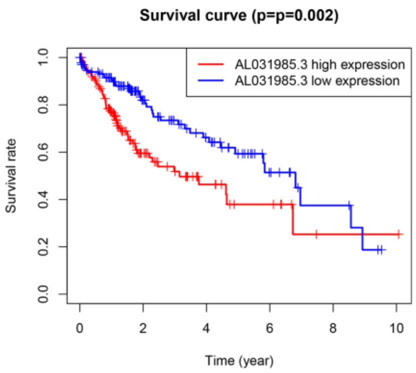

(C)

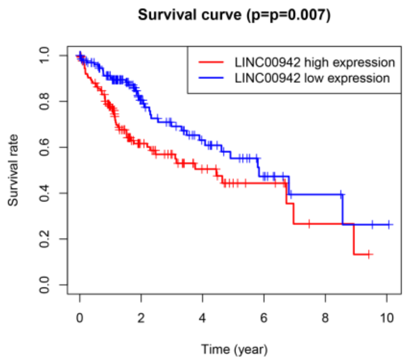

(D)

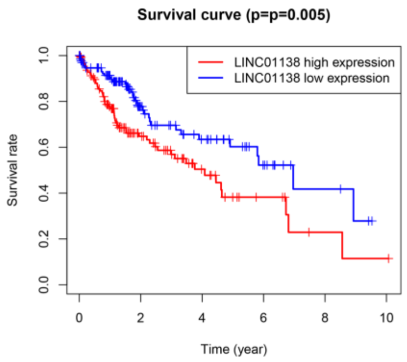

(E)

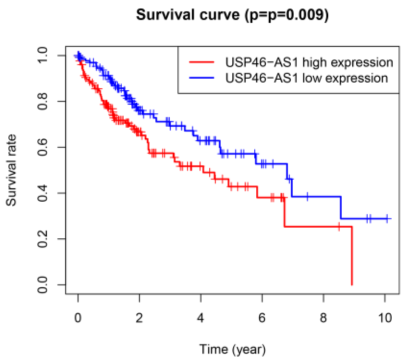

Supplement: Supplementary Files S1-S2 [file BSR-2020-4442_supp.pdf]
